# Performance of the Abbott SARS-CoV-2 IgG II Quantitative Antibody Assay Including the New Variants of Concern, VOC 202012/V1 (United Kingdom) and VOC 202012/V2 (South Africa), and First Steps towards Global Harmonization of COVID-19 Antibody Methods

**DOI:** 10.1128/JCM.00288-21

**Published:** 2021-08-18

**Authors:** Emma English, Laura E. Cook, Isabelle Piec, Samir Dervisevic, William D. Fraser, W. Garry John

**Affiliations:** a Faculty of Medicine and Health, University of East Anglia, Norfolk, United Kingdom; b Department of Clinical Biochemistry and Immunology, Norfolk and Norwich University Hospital, Norfolk, United Kingdom; c Department of Virology, Norfolk and Norwich University Hospital, Norfolk, United Kingdom; Cepheid

**Keywords:** COVID-19, SARS-CoV-2, antibody assay, serology, evaluation, harmonization, variants, analytical performance

## Abstract

In the initial stages of the severe acute respiratory syndrome coronavirus 2 (SARS-CoV-2) COVID-19 pandemic, a plethora of new serology tests were developed and introduced to the global market. Many were not evaluated rigorously, and there is a significant lack of concordance in results across methods. To enable meaningful clinical decisions to be made, robustly evaluated, quantitative serology methods are needed. These should be harmonized to a primary reference material, allowing for the comparison of trial data and improved clinical decision making. A comprehensive evaluation of the new Abbott IgG II anti-SARS-CoV-2 IgG method was undertaken using CLSI-based protocols. Two different candidate primary reference materials and verification panels were assessed with a goal to move toward harmonization. The Abbott IgG II method performed well across a wide range of parameters with excellent imprecision (<3.5%) and was linear throughout the positive range (tested to 38,365 AU/ml). The sensitivity (based on ≥14-day post-positive reverse transcription-PCR [RT-PCR] samples) and specificity were 98.3% (90.6% to 100.0%) and 99.5% (97.1% to 100%), respectively. The candidate reference materials showed poor correlation across methods, with mixed responses noted in methods that use the spike protein versus the nucleocapsid proteins as their binding antigen. The Abbott IgG II anti-SARS-CoV-2 measurement appears to be the first linear method potentially capable of monitoring the immune response to natural infection, including from new emerging variants. The candidate reference materials assessed did not generate uniform results across several methods, and further steps are needed to enable the harmonization process.

## INTRODUCTION

Severe acute respiratory syndrome coronavirus 2 (SARS-CoV-2) has swept the globe at an alarming rate, with a reported 90 million cases and 1.9 million deaths by the 1-year anniversary of the first death from this pandemic (1) and increasing to 119 million cumulative cases and >2.6 million deaths by the middle of March 2021 (2). In this time, there has been an unprecedented global effort to identify new diagnostic tests, treatments, and, more recently, vaccines against the virus and the associated disease, COVID-19.

The development and delivery of a range of vaccines against the virus is under way, with several already approved for use and others in late-stage clinical trials (3). Although there are several different approaches to the design of these vaccines, a common factor is the use of the spike proteins, in the form of the attenuated whole virus, or portions of the spike protein such as the receptor binding domain or through the use of nucleic acids directed to the synthesis of the spike protein.

In the United Kingdom, a national immunization program has been launched, with a tiered system of invitations to receive the vaccine dependent on risk of a negative outcome from the disease (4). While there is trial data for each of the vaccines in use, there are limited data regarding the quantitative changes in antibody concentrations over time following vaccination. With the introduction of a national immunization program, it will be important to understand the antibody response to immunization in terms of development, peak concentration, and decline over time to assess efficacy of the vaccination delivery. To do this, two elements are required: a robust quantitative SARS-CoV-2 IgG method, which is directed against the spike protein, and a commutable standard or reference material to allow comparison of results across different methods and thus different trials or immunization programs (5).

This study evaluated several assay performance criteria (such as precision and sensitivity) using recognized and standardized evaluation protocols (EPs) for the new Abbott SARS-CoV-2 IgG II Quant method on the Alinity i system (Abbott Diagnostics, Chicago, IL, USA) and explored the different materials available that may form the basis of a candidate international reference standard for harmonization programs (6).

## MATERIALS AND METHODS

### Sample collection and storage.

All procedures were performed in accordance with the ethical standards of University of East Anglia for deidentified samples for method development and in concordance with the Helsinki Declaration. Serum samples were collected, anonymized, aliquoted, and stored at −80°C until analyzed. SARS-CoV-2-positive samples (P) were from patients with PCR-confirmed infection (AusDiagnostics platform [Chesham, UK], the Panther [Manchester, UK], and Altona [Hamburg, Germany]). All reverse transcription-PCR (RT-PCR) assays have dual genome targets. Key performance testing, including precision, limit of quantitation (LOQ), linearity, and method comparison, were assessed per Clinical and Laboratory Standards Institute (CLSI) protocols, which ensured standardized testing procedures were used.

### Summary of the Abbott assay.

The SARS-CoV-2 IgG II Quant assay is an automated, two-step chemiluminescent microparticle immunoassay (CMIA). It is used for the qualitative and quantitative determination of IgG antibodies to the receptor binding domain (RBD) of the S1 subunit of the spike protein of SARS-CoV-2 in human serum and plasma on the Alinity i system. The sequence used for the RBD was taken from the WH-Human 1 coronavirus, GenBank accession number MN908947. The analytical measurement interval is stated as 21 to 40,000 AU/ml, and positivity cutoff is ≥50 AU/ml (manufacturer defined).

### CLSI EP-5 and EP-15 imprecision.

Both CLSI EP-5- and EP-15-based protocols were used to evaluate the imprecision of the assay. For the EP-15 study, three different quality control (QC) levels (Abbott Diagnostics) were used (one negative and two positives), and five replicates were measured twice a day for 5 days. For the EP-5 study, four patient serum pools (one negative and 3 different positives) were measured in duplicates twice a day for 20 days.

### CLSI EP-6 linearity.

Dilutions of a high-titer patient sample (mean value, 38,365 arbitrary units [AU]/ml, from triplicate measurements) were made using the Abbott diluent to generate a series of samples with antibody concentrations over 95% of the analytical measurement range of the assay. All samples were measured in triplicates.

### Limit of quantitation and limit of detection.

The LOQ was determined by measuring five negative patient pools in quintuplicates twice a day for 2 days. The LOQ was estimated as the lowest concentration with a 20% coefficient of variation (CV) (7). As defined in CLSI EP-17, LOD is determined by utilizing both the measured limit of the blank (LOB) and test replicates of a sample known to contain a low concentration of analyte using the equation LOD = LOB + 1.645 × SD_low-concentration sample_, where SD is the standard deviation (7) The Abbott diluent was used to determine the LOB, as it is the diluent used for on-board dilution.

### Cross-reactivity samples.

Negative-control samples were from healthy patients with no recorded history of infection or immune disorders and collected in 2018, prior to the emergence of COVID-19. Prepandemic samples from patients who had a range of confirmed respiratory infections (including influenza A and B and seasonal coronaviruses) were included in the cross-reactivity analysis. Samples from patients positive for thyroid stimulating immunoglobulin (TSI) were analyzed to test the nonspecific binding of non-SARS-CoV-2 antibodies in the assay. These groups of samples are referred to as N (negative control), CR (cross-reactivity), and TSI (patients with thyroid stimulating immunoglobulin); for further details on samples and collection, please see reference 8. A total of 334 individual serum samples (143 P, 65 N, 97 CR, and 29 TSI) were analyzed for SARS-CoV-2 IgG antibodies.

### Specificity and sensitivity analysis.

The quantitative IgG levels were measured at different time points after a confirmatory RT-PCR test for SARS-CoV-2, allowing for analysis at pre- and post-14 days from the RT-PCR date; along with the cross-reactivity samples, these were used to determine sensitivity and specificity.

A concordance analysis was undertaken comparing the Abbott quantitative method with 3 other SARS-CoV-2 IgG immunoassay methods: (i) Epitope Diagnostics Inc. (EDI, San Diego, CA, USA) performed an automated Agility enzyme-linked immunosorbent assay (ELISA) (Dynex Technologies, Chantilly, VA, USA), (ii) Abbott Diagnostics (Maidenhead, UK) utilized a qualitative method on the Alinity i analyzer, and (iii) DiaSorin (Dartford, UK) utilized the Liaison XL analyzer.

### New variant samples.

As the pandemic progresses, new variants of the virus emerge, raising concern that the mutations in these variants may render immunoassays ineffective, as the antigenic changes that arise may no longer represent the antigenic regions of the reagents in the assay. The main SARS CoV-2 lineage circulating in the autumn of 2020 in the United Kingdom was B.1.177 (the Spanish lineage). However, since January 2021, the “UK variant” (VOC 202012/V1 or B1.1.7) has become the predominant virus in the United Kingdom (9). Furthermore, by the beginning of March 2021, there have been in total 266 confirmed and probable cases of a “South Africa” variant (VOC 202012/V2 (B.1.351). These new variants have several mutations in different parts of SARS CoV-2 genome, with some within the receptor binding domain increasing the virus transmissibility. The RBD of the “UK” (VOC 202012/V1 or B1.1.7) strain, which is the predominant virus in the United Kingdom, contains mutation N501Y in the RBD domain of the spike protein among 15 other mutations in other genome areas. This variant has, since January 2021, acquired another RBD mutation, E484K, in addition to the variant-defining mutations, which resulted in its designation as VOC 202102/02 (B1.1.7 cluster with E484K). E484K is currently the mutation with most evidence of causing antigenic change (10). The RBD of the “South Africa” (VOC 202012/V2 or B.1.351) variant contains an RBD K417N mutation in subsets of isolates in addition to the E484K and N501Y RBD mutations.

Viruses containing the above-described mutations are not very similar to the predominant virus (B.1.177) in circulation in the summer and autumn of 2020.

We analyzed samples from patients proven to have the VOC 202012/0V1 (UK) strain, which is now a predominant virus in the United Kingodm, as well as one imported case of the VOC 202012/V2 (South Africa) strain.

### CLSI EP-9 (trueness).

Although a small number of assays are marketed as quantitative methods, there are no standardized reporting units, making method comparisons difficult. It was considered inappropriate to evaluate trueness using a standard CLSI EP-9 protocol. See the section below for further discussion.

### Identifying potential standards and reference materials.

To progress the harmonization of SARS-CoV-2 Ig immunoassay methods, a certified reference material (CRM) is needed. Several candidate standard materials were evaluated on each of the four methods described above. Three different materials were obtained from the National Institute for Biological Standards and Control (NIBSC) and assayed.

• One was a CE-marked verification panel of 37 samples (NIBSC code 20/B770). Each sample consisted of 0.3 ml of human plasma containing the bacterial growth inhibitor Bronidox at 0.05% (wt/vol). Twenty-three samples were convalescent plasma packs known to be anti-SARS-CoV-2 positive, and the remaining 14 were detailed as negative.
• The second was a CE-marked “working standard” (NIBSC code 20/162) intended for use as a diagnostic calibrant to monitor the sensitivity of assays. The standard consisted of convalescent plasma positive for anti-SARS-CoV-2 antibodies pooled from three different donors. Frozen liquid (0.3 ml) was supplied. The material has been assigned an arbitrary unitage of 1,000 U. A series of dilutions were made to assess the linearity at the positive cutoff value.
• The third was CE-marked “quality control 1” (NIBSC code 20/B764) intended for internal quality control use for immunoassays that detect SARS-CoV-2 antibodies. The material was supplied as a ready-to-use reagent of plasma positive for anti-SARS-CoV-2 antibodies, derived from two different donors and diluted in defibrinated convalescent plasma and preserved with Bronidox at 0.05% (wt/vol).

A panel of heat-inactivated “reference materials” with 5 positive samples and 1 negative sample from Technopath were assayed (Technopath, Tipperary, Ireland). The manufacturer’s information suggests the samples are a series of prediluted samples from a positive stock. The primary material, the diluent used, and the heat inactivation process were not described.

### Statistics.

Calculations were performed using SPSS Statistics (IBM) 25.0.0.1. or GraphPad Prism version 8.0 (GraphPad Software, Inc., USA). Cohen’s kappa tests were used to determine the concordance between the assays. Analysis of CLSI EP-15 was performed using the software EP evaluator (Data Innovations, build 11.3.0.23).

## RESULTS

### CLSI EP-5 and EP-15 imprecision.

Table S1 in the supplemental material shows the performance of a negative and two positive QC samples using the CLSI EP-15-based protocol. The mean value of the negative sample was very low (3.4 AU/ml) and outside the analytical measurement range, hence the high CV at that level; however, the SD value was low (0.7), inferring good performance. The imprecision of the positive QC materials was low (3.0% and 3.3% total CV).

The imprecision data based on CLSI EP-5 is as follows. There was a high CV in the negative samples (CV, 64%; mean, 2.7 AU/ml; SD, 1.74 AU/ml), but the more relevant total CV in each of the three positive samples remained below 3.5%, with 2.9%, 3.3%, and 3.4% for pool 1 (mean, 71 AU/ml; SD, 2.09 AU/ml), pool 2 (mean, 283 AU/ml; SD, 9.3 AU/ml), and pool 3 (mean, 2,428 AU/ml; SD, 83.4 AU/ml), respectively.

### CLSI EP-6 linearity.

**FIG 1:**
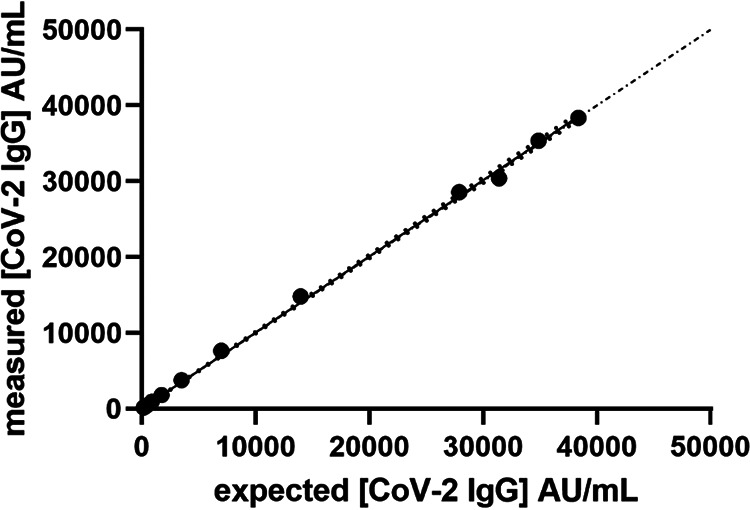
Linearity of method over the complete working range of the Abbott IgG II assay using a range of dilutions of a high positive (mean, 38,365 AU/ml) in the Abbott diluent. Dash-dot line indicates the identity line. The darker dotted line represents the 95% likelihood asymmetrical CI of the slope.

CLSI states, for EP-6, that goals for linearity should be derived from goals for bias and should be less than or equal to these goals. Fig. 1 shows the linearity of a diluted sample over the working range of the assay, it is linear up to 38,365 AU/ml as tested (manufacturer claim is 40,000 AU/ml). This was the highest patient sample value available measured neat that was under 40,000 AU/ml and was acceptable, as this high-value sample was within 5% of the upper limit. Table S2 details the percent difference from the target values for each dilution. Linear fitting was performed and showed a slope of 1.004 (95% confidence interval [CI], 0.9923 to 1.017), with an *r*^2^ of 0.9992. The model was tested and returned a *P* value of <0.001, indicating nondeviation from linearity.

### Limit of quantitation and limit of detection.

The means and the CVs (%) of the samples for the LOQ were 3.8 AU/ml (59.3%), 18.1 AU/ml (13.6%), 30.4 AU/ml (5.4%), 36.9 AU/ml (3.4%), and 52.2 AU/ml (3.9%). The LOQ was estimated as the lowest concentration with a 20% CV. Using a four-parameter logistic (4-PL) curve fit, the LOQ was calculated at 15.4 AU/ml. As defined in CLSI EP-17, LOD is determined by utilizing both the measured LOB (0.1 AU/ml) (lower limit of blank) and test replicates of a sample known to contain a low concentration of analyte. The LOD was determined as 4.3 AU/ml using the Abbott diluent.

### Cross-reactivity samples.

Patient samples (*n* = 97) from people with respiratory infections, collected in 2018 and 2019 prior to the COVID-19 pandemic, were analyzed. The results ranged from 1.1 AU/ml to 48.3 AU/ml, with a mean value of 6.9 AU/ml. All but one sample were reported as negative, but one was also close to the cutoff of 50 AU/ml (manufacturer defined). One sample had a value of 140.5 AU/ml but was negative on the qualitative IgG assay and the IgM assay. In addition to these CR samples, 29 TSI samples were also analyzed, and all returned negative values from 0.0 to 29.0 AU/ml. Data are summarized in Table 1.

**TABLE 1:** Summary of the sensitivity, specificity, and cross-reactivity samples of Abbott IgG II quantitative anti-SARS-CoV-2 IgG method

Group	No. of samples			Result (% [95% CI])
	Total	SARS-CoV-2 IgG positive	SARS-CoV-2 IgG negative	
SARS-CoV-2-positive samples				
All time points	143	131	12	91.6 (85.8–95.6)
>14 days	57	56	1	98.3 (90.6–100.0)
Pre-COVID-19 controls (N)	65	0	65	100.0 (94.5–100.0)
Other respiratory infections (CR)	97	1	96	99.0 (94.5–100.0)
Thyroid stimulating immunoglobulin (TSI)	29	0	29	100.0 (88.1–100.0)
Controls (N, CR, and TSI)	191	1	190	99.4 (97.1–100.0)

### Specificity and sensitivity.

Table 1 also details the sensitivity and specificity of the method with analysis of SARS-CoV-2 RT-PCR-positive samples at all time points and at >14 days post-confirmatory test. The data show that the method has a sensitivity of 91.6% in all time points and 98.3% at >14 days and a specificity of 99.4%.

**FIG 2:**
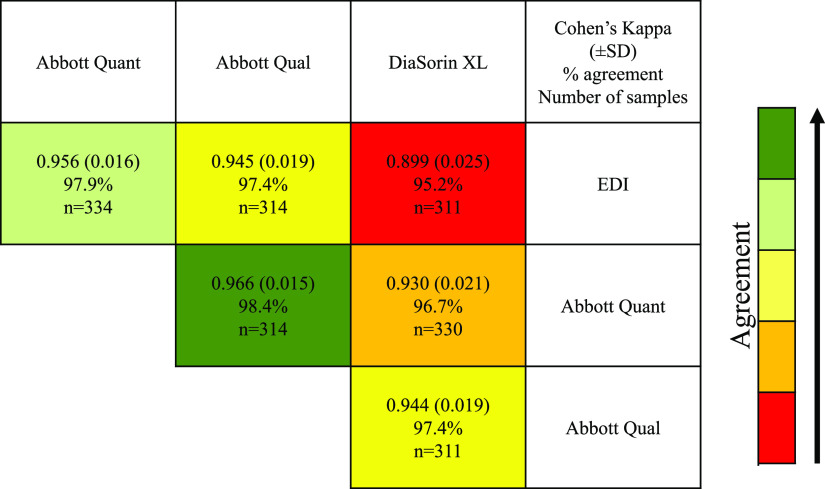
Cohen’s kappa concordance analysis of the assays and overall (all samples included) agreement of results given as percent. Equivocal results were considered negative.

The analysis of assay concordance revealed a mixed pattern of agreement, with the highest between the Abbott quantitative method (IgG II) and the Abbott qualitative method, with a Cohen’s kappa of 0.965 and agreement of 98.4%, and the poorest between the DiaSorin and other methods (Cohen’s kappa of 0.930, agreement of 96.7% between the Abbott quantitative and the DiaSorin methods) (see Fig. 2).

### New variant samples.

**FIG 3:**
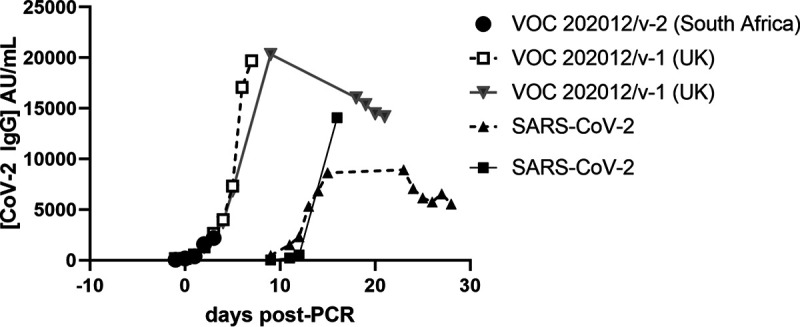
Representative examples of the quantitative immune response in three different variants of the SARS-CoV-2 virus, including the “UK” and “South Africa” variants. The days post-PCR do not necessarily correlate to the day of onset of symptoms or the day of hospitalization.

The results clearly show that the Abbott IgG II method detects the original strain of SARS-CoV-2 as well as two new variants of concern, the VOC 202012/V1 (UK) strain and the VOC 202012/V2 (South Africa) strain. Fig. 3 shows a time course for a subset of 4 different patients, charting the increase in antibody levels post-confirmatory RT-PCR.

### Identifying potential standards.

The analysis of the 37 samples from the NIBSC “verification panel” produced the expected classification of 23 positive and 14 negative samples, with a clear separation between the two groups on the Abbott IgG II assay (see Table S3). The range of the positive values was 210 to 9,710 AU/ml. The values for the negative samples of 0.7 to 5.6 AU/ml were significantly below the ≥50-AU/ml threshold for classification as a positive sample. As expected, these values are markedly different from the values presented for other methods. To allow comparison of the results between the different methods and to standardize the results, the individual values were normalized to the highest responding sample for each method, which was set to a normalizing value of 1. Thus, the results of the positive samples for each method were divided by the highest value obtained by that method for any of the positive 23 samples. So, if the highest positive values were 50 and 23 for two methods, all positive samples were divided by 50 for the first method and by 23 for the second method. This provided all values as a ratio of the highest value obtained for any sample on an individual method. This should demonstrate if the magnitudes of positive response compare between methods (see Fig. S1). Fig. 1 shows that methods which use spike proteins as the assay antigen produced similar results. Methods that used the nucleocapsid antigen were similar. However, there is a lack of agreement between these two method types (spike Ag versus nucleocapsid Ag).

The analysis of the NIBSC working standard (NIBSC code 20/162) and quality control 1 (NIBSC code 20/B764) samples generated mean values of 14,072 AU/ml and 296.6 AU/ml, respectively, on the Abbott IgG II method, as means from triplicates. This is significantly different from the arbitrary “1,000 U” assigned to the working standard. It should be noted that no volume was detailed in the unit assignment. The dilution of the NIBSC 20/162 working standard proved linear. The slope was 0.9981 (95% CI, 0.9836 to 1.013), with an *r*^2^ of 0.9997. The model test showed no deviation from the linear model, with a *P* value of 0.2500. The percent difference from expected is detailed in Table S4; the range was 4.6% to 9.7% difference to a value of 20 AU/ml on dilution.

**FIG 4:**
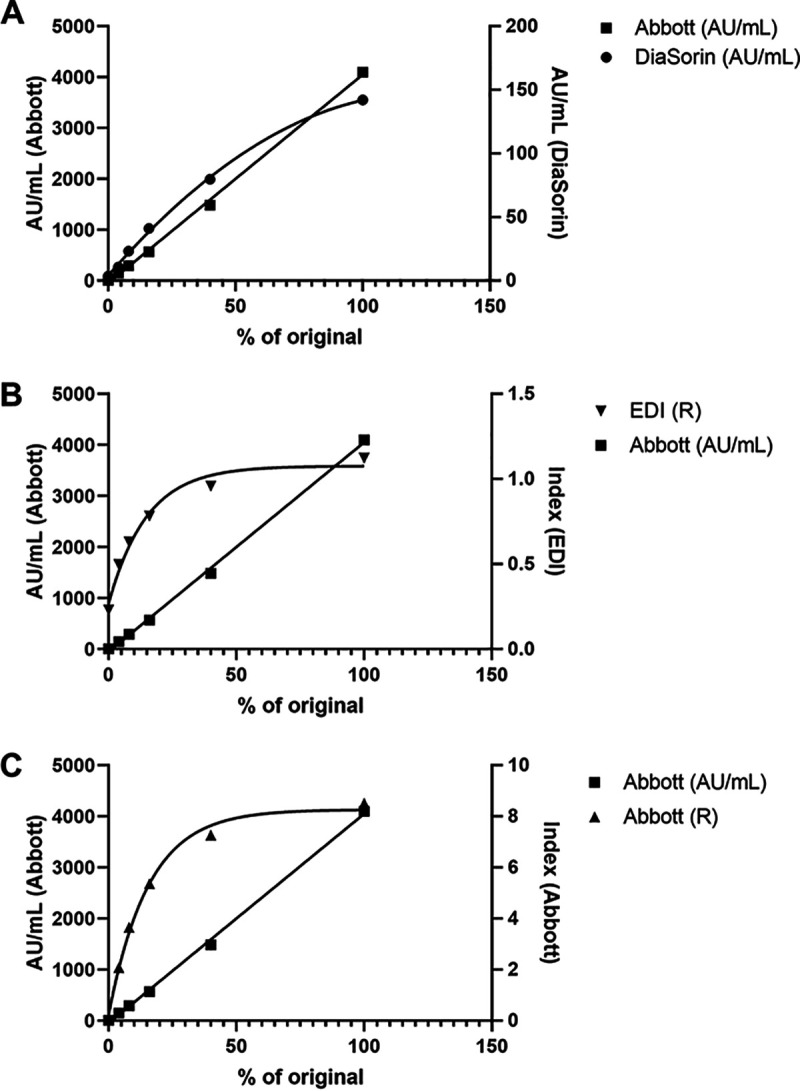
Comparison graphs of the values obtained for the Technopath positive panel with different methods: (A) Abbott IgG II versus DiaSorin Liaison XL; (B) Abbott IgG II versus EDI; (C) Abbott IgG II quantitative (S) versus Abbott IgG qualitative (R). Only the Abbott quantitative assay showed linearity (*r*^2^ = 0.9984) and was plotted against DiaSorin, quadratic (*r*^2^ = 0.9988) (A), EDI, 4-PL (*r*^2^ = 0.9574) (B), and Abbott qualitative, 4-PL (*r*^2^ = 0.9946) (C).

**FIG 5:**
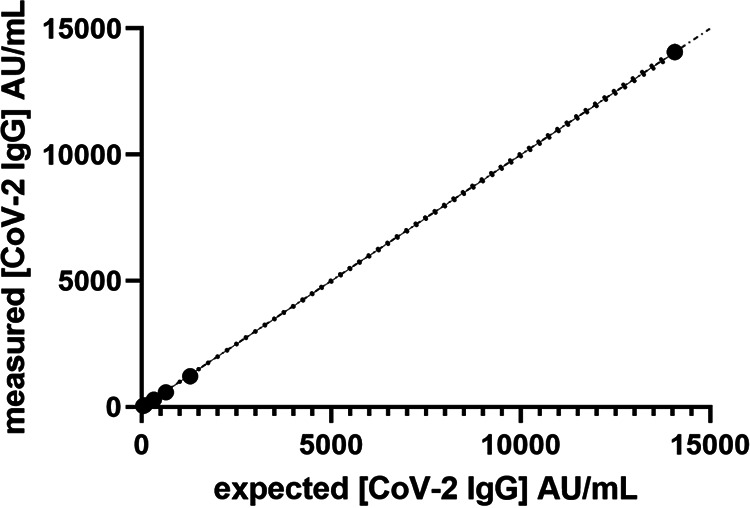
Dilution of NIBSC working standard 20/162 using the Abbott diluent. Dash-dot line indicates the identity line. The darker dotted line represents the 95% likelihood asymmetrical CI of the slope.

The Technopath series proved to be linear when measured by the quantitative method; this was expected, as the samples represent a dilution series. Although the diluent was not described, it does not appear to have had an impact on the linearity of the dilution series. The range of values obtained were 5.6 AU/ml for the negative and 147.5 to 4,098 AU/ml for the positive samples. Figure 4 shows comparison graphs of the values obtained with the Abbott IgG II versus those with different methods (DiaSorin Liaison XL, EDI, Abbott IgG [qual]). The other methods are clearly calibrated toward the negative/positive threshold, and were not linear using this material. Figure 5 shows the dilution of NIBSC working standard 20/162 using the Abbott diluent.

## DISCUSSION

### Quantitative SARS-CoV-2 IgG II method evaluation.

The focus of many method evaluations to date has been on the diagnostic accuracy of the assay; does it correctly identify those with or without antibodies to the SARS-CoV-2 virus? However, many publications have neglected to fully address the analytical performance of these methods, which ultimately has a significant impact of the potential clinical utility of these tests. There are >300 different methods in development or available for purchase that aim to detect SARS-CoV-2 IgG, and while some have undergone a robust evaluation, many on the market have not (11, 12). This is the first evaluation of the prelaunch Abbott anti-SARS-CoV-2 IgG assay (IgG II) on the Alinity i system, and the data clearly show that it meets many of the expected performance criteria.

The method achieved an excellent precision profile, which is well within the ≤5% CV often cited as the minimum criteria set by the Food and Drug Administration (FDA) and the European Medicines Agency (EMA) (13). The method is linear across a wide working range. The sensitivity and specificity are very high, at 98.3% (90.6% to 100%) for samples ≥14 days post-positive RT-PCR and 91.6% (85.8% to 95.6%) for all samples and 99.5% (97.1% to 100%), respectively. Although concordance with other methods varied, it is important to note that this is the first quantitative assay for SARS-CoV-2 IgG; therefore, it is difficult to make direct comparisons. The poorest concordance was with the DiaSorin method, reflecting previously published data (8, 14, 15).

It is reassuring to see that the method also identifies antibodies in patients with the two new variants of concern (VOC 202012/V1 [UK] and VOC 202012/V2 [South Africa] strains) and that an increase in antibody levels occurs as the immune response evolves. It is important that any method used to monitor immune response to infection is able to detect antibodies that arise from a variety of emergent variants; otherwise, false-negative diagnoses may arise.

### Identification of a candidate primary reference material.

A robust approach to harmonization of serology methods is essential in order to understand the ongoing impacts of both natural infection and vaccination on the immunity of the population to SARS-CoV-2. This study and our previous data (8) have shown a significant disparity in the performance of different commercial methods in terms of linearity (Fig. 4), units of measurement (Fig. 4), and even sensitivity and specificity (Fig. 2, Table 1, and reference 8).

This study evaluated candidate reference materials for the harmonization of anti-SARS-CoV-2 antibody methods. Much hope has been placed on the rapid introduction of vaccines against this virus, but many questions on their efficacy remain unanswered. Questions such as are two doses of the vaccine needed? What time interval is most effective? What is the magnitude and duration of the immune response? What level of antibodies in circulation are needed to continue to afford protection? All these questions require multiple, large-scale, and multisite studies to answer, which in return need robust and consistent serology measurements. Studies in children who have been vaccinated for Rubella virus show that approximately 9% are seronegative after the first dose, decreasing to <1% after the second, clearly indicating the value of the second dose (16, 17). Antibody levels present in a population are higher when due to naturally occurring immunity or postvaccination boosts from virus exposure than levels that arise through immunization alone, meaning the expected values for Rubella virus antibodies have decreased as immunization programs have widened their reach (17); this may be mirrored over time with the SARS-CoV-2 antibody levels in the general population, meaning that any derived target values for positive serology and the limits of quantitation of methods will need to adapt over time.

To achieve the goal of harmonized serology testing for anti-SARS-CoV-2 antibody methods, the principles of metrology must be applied (18–20). Key components of a system of traceability include a defined measurand, a primary reference material (preferably approved by a certifying authority such as IFCC, ISO, ICM, NIST, etc.), a higher-order measurement system or reference method procedure, and a known calibration hierarchy. Metrological traceability is the property of a measurement result, whereby the result can be related to a reference through a documented unbroken chain of calibrations, each contributing to the measurement uncertainty (19). While this has been achieved for analytes such as HbA1c, there has been less success with serology testing for viruses such as Rubella virus and, to date, very limited attempts for anti-SARS-CoV-2 antibody methods (17, 21, 22).

Hurdles to overcome include the availability of a reference measurement procedure and a primary reference material. The immune response to an antigen challenge is heterogenous; therefore, defining the measurand is difficult. Three components, comprising the system (or matrix such as plasma), the component (the anti-SARS CoV-2 IgG), and a measurement quantity such as the biological response or biological activity, together form the measurand of interest. It is expected that a primary reference material for such complex analytes will undergo state-of-the-art purification steps, with identification of class and subclass of immunoglobulin and some type of functional assessment of biological activity. International units per milliliter (IU/ml) should be used, and the reference material should be commutable across methods (6, 23). Once a primary reference material has been defined, all future reference materials should refer to this material rather than to the previous batch.

This standardization process has proven difficult for serology methods, with Rubella virus IgG methods an example of how poor the agreement is between some methods (24). This has the potential to lead to misinterpretation of results, sometimes causing adverse clinical outcome. Factors which influenced the lack of standardization include the use of an immunoglobulin preparation from human serum, with limited purification steps, which are not described. The effect on biological activity of the preparation, lyophilization, and subsequent reconstitution was not assessed. Guidance on appropriate diluents was not provided. Similarly, these are the same conditions under which the current available reference materials for SARS-CoV-2 antibody methods are prepared, and the same questions around performance are being raised.

Our data show that the current anti-SARS-CoV-2 antibody methods do not compare well in terms of units of measurement, linearity, magnitude of response, and relative response in different patient samples. Those methods which detect antibodies directed against the spike protein appear to have greater concordance with each other than those that detect the nucleocapsid. Some of this may be due to the calibration of the methods, with only the Abbott IgG II quantitative method being linear so far. The materials currently available as candidate primary reference materials show considerable variation across methods, and the preparation and performance of these materials are poorly described. Without steps to improve the quality of these reference materials, including a description of the antibody populations within the reference material (i.e., predominantly nucleocapsid or spike protein recognition), the scientific community is likely to encounter similar pitfalls to those that previous attempts to harmonize serology methods have experienced.

### Conclusions.

It is clear to see that there is a long road ahead to achieve harmonization of anti-SARS-CoV-2 antibody methods, and urgent action is needed to ensure that manufacturers and regulatory bodies work synergistically toward the goal of harmonization.

The Abbott IgG II method performed well in this evaluation and is the only method tested that shows linearity over a wide concentration range and potential external calibration materials. It is suitable for future studies investigating the clinical response to natural infection, which are urgently needed.
