# Supplementary material for: Performance of the Abbott SARS-CoV-2 IgG II Quantitative Antibody Assay Including the New Variants of Concern, VOC 202012/V1 (United Kingdom) and VOC 202012/V2 (South Africa), and First Steps towards Global Harmonization of COVID-19 Antibody Methods
Source: J Clin Microbiol. 2021 Aug 18;59(9):e00288-21. doi: 10.1128/JCM.00288-21 (PMC8373017; doi:10.1128/JCM.00288-21)

Supplemental Table 1: EP-15 (5 day imprecision data) generated using Abbott QC material

	Negative QC Target (N/A) range (N/A-44.5 AU/mL)		Positive 1 Target (154.6 AU/mL) range (92.8-201.0 AU/mL)		Positive 2 Target (766.0 AU/mL) range (459.6-995.8 AU/mL)	
	Mean 3.4 AU/mL		Mean 162.9 AU/mL		Mean 604.2 AU/mL	
	%CV	SD	%CV	SD	%CV	SD
**Total**	**20.5**	0.7	**3.0**	4.9	**3.3**	19.7

Supplemental Table 2: Expected, measured and % difference values for the assessment of linearity of the Abbott IgG II assay using a range of dilutions of a high positive (mean 38,365 AU/mL) in the Abbott diluent.

Expected Values (AU/mL)	Measured Values (AU/mL)	% Difference
38365	38365	0
34877	35343	1
31389	30392	-3
27902	28553	2
13951	14810	6
6975	7646	10
3488	3770	8
1744	1795	3
872	926	6
436	422	-3
327	326	0
218	223	2
163	164	1
109	122	12
82	90	10
68	74	9
41	50	23
34	40	19
27	32	18

Supplemental Table 3: Results obtained on the Abbott SARS-CoV-2 IgG II quantitative method for the 37 samples of the NIBSC 'verification panel'. Samples were measured in duplicate

Panel Number	Result replicate 1 (AU/mL)	Result replicate 2 (AU/mL)	Average (AU/mL)	Positive or Negative
1	211.0	209.1	210.1	P
2	348.4	339.6	344.0	P
3	8707.3	8559.6	8633.5	P
4	6260.3	6384.1	6322.2	P
5	9710.5	9708.5	9709.5	P
6	4225.2	3974.8	4100.0	P
7	2304.3	2147.7	2226.0	P
8	4428.8	4011.8	4220.3	P
9	6429.8	6383.6	6406.7	P
10	6674.9	7115.1	6895.0	P
11	1172.3	1186.2	1179.3	P
12	1167.6	1127.9	1147.8	P
13	1395.3	1382.3	1388.8	P
14	961.6	999.0	980.3	P
15	1590.9	1685.6	1638.3	P
16	787.6	747.4	767.5	P
17	2617.7	2541.6	2579.7	P
18	6062.8	6065.6	6064.2	P
19	2329.8	2355.0	2342.4	P
20	1920.4	1954.4	1937.4	P
21	3111.3	3117.9	3114.6	P
22	3517.7	3447.6	3482.7	P
23	2290.0	2314.6	2302.3	P
24	4.0	2.0	3.0	N
25	4.7	4.2	4.5	N
26	1.9	3.8	2.9	N
27	3.4	2.4	2.9	N
28	0.8	1.0	0.9	N
29	2.0	1.3	1.7	N
30	5.6	3.2	4.4	N
31	1.6	1.3	1.5	N
32	0.7	3.0	1.9	N
33	0.6	3.1	1.9	N
34	1.6	1.5	1.6	N
35	0.9	0.4	0.7	N
36	2.3	2.3	2.3	N
37	5.1	0.6	2.9	N

Supplemental Table 4: Expected, measured and % difference values for the assessment of linearity of the Abbott IgG II assay using a range of dilutions of the NIBSC 20/162 working standard in the Abbott diluent.

Expected Values (AU/mL)	Measured Values (AU/mL)	% Difference
14072.4	14072.4	0
1279.3	1220.8	-4.56
639.6	580.3	-9.3
319.8	288.8	-9.7
80.0	74.8	-6.5
40.0	38.1	-4.7
20.0	18.5	-7.6
4.0	3.3	-17.7

Supplemental Figure 1: Comparison of concordance of different methods using the NIBSC 37 sample verification panel. Graph shows the results of the positive samples from the verification panel from NIBSC for (A) spike protein-based Abbott Quantitative and DiaSorin (AU/mL) assays and (B) nucleocapsid-based Abbott qualitative and EDI assays (index, R). Original data were normalised as a ratio to the highest value for each assay and ordered from the lowest to the highest values obtained in the Abbott quantitative assay.

A

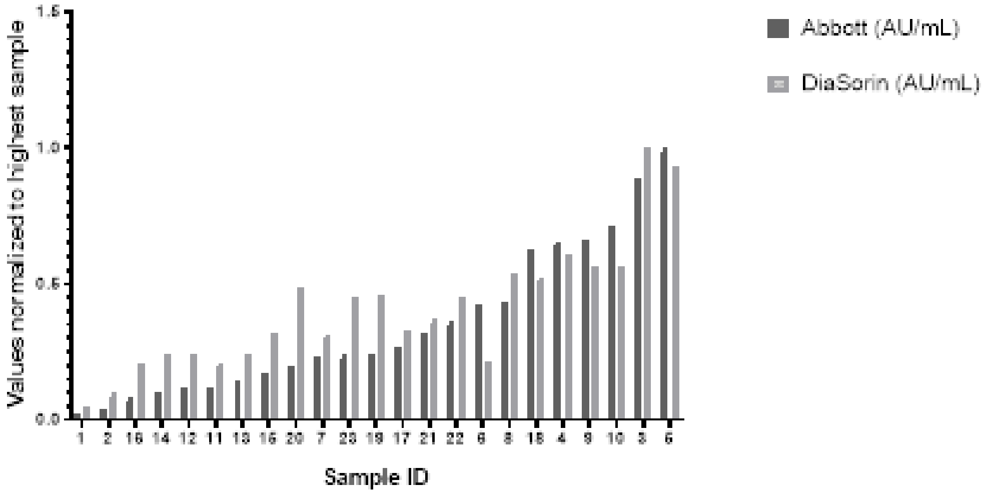

B

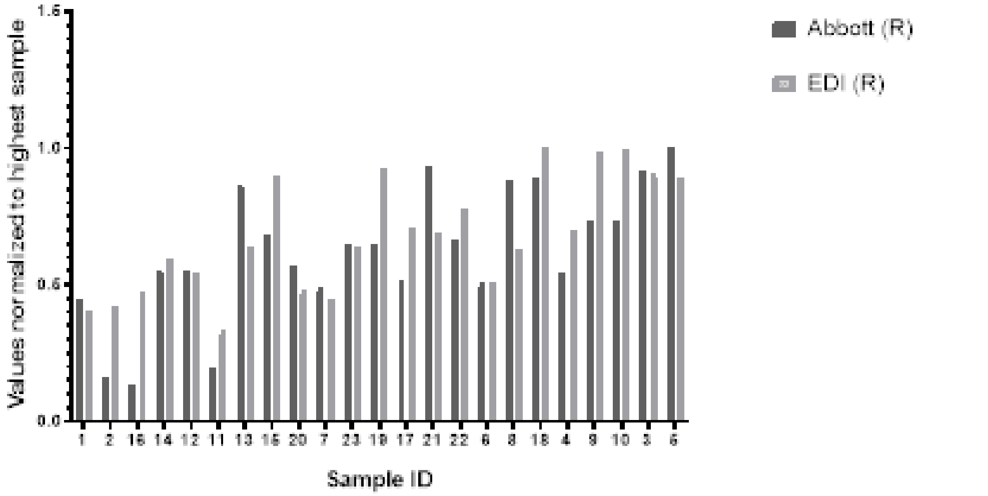

Supplement: Supplemental file 1 — Tables S1 to S4 and Fig. S1. Download JCM.00288-21-s0001.pdf, PDF file, 0.2 MB [file jcm.00288-21-s0001.pdf]
